# Early uneven ear input induces long-lasting differences in left–right motor function

**DOI:** 10.1371/journal.pbio.2002988

**Published:** 2018-03-13

**Authors:** Michelle W. Antoine, Xiaoxia Zhu, Marianne Dieterich, Thomas Brandt, Sarath Vijayakumar, Nicholas McKeehan, Joseph C. Arezzo, R. Suzanne Zukin, David A. Borkholder, Sherri M. Jones, Robert D. Frisina, Jean M. Hébert

**Affiliations:** 1 Department of Neuroscience, Albert Einstein College of Medicine, Bronx, New York, United States of America; 2 Departments of Chemical & Biomedical Engineering and Communication Sciences & Disorders, Global Center for Hearing & Speech Research, University of South Florida, Tampa, Florida, United States of America; 3 Department of Neurology, Ludwig-Maximilians University Munich and Munich Cluster for Systems Neurology (SyNergy), Munich, Germany; 4 Institute for Clinical Neurosciences, Ludwig-Maximilians University Munich, Munich, Germany; 5 Department of Special Education and Communication Disorders, University of Nebraska-Lincoln, Lincoln, Nebraska, United States of America; 6 Microsystems Engineering, Rochester Institute of Technology, Rochester, New York, United States of America; 7 Department of Genetics, Albert Einstein College of Medicine, Bronx, New York, United States of America; Robarts Research Institute, Canada

## Abstract

How asymmetries in motor behavior become established normally or atypically in mammals remains unclear. An established model for motor asymmetry that is conserved across mammals can be obtained by experimentally inducing asymmetric striatal dopamine activity. However, the factors that can cause motor asymmetries in the absence of experimental manipulations to the brain remain unknown. Here, we show that mice with inner ear dysfunction display a robust left or right rotational preference, and this motor preference reflects an atypical asymmetry in cortico-striatal neurotransmission. By unilaterally targeting striatal activity with an antagonist of extracellular signal-regulated kinase (ERK), a downstream integrator of striatal neurotransmitter signaling, we can reverse or exaggerate rotational preference in these mice. By surgically biasing vestibular failure to one ear, we can dictate the direction of motor preference, illustrating the influence of uneven vestibular failure in establishing the outward asymmetries in motor preference. The inner ear–induced striatal asymmetries identified here intersect with non–ear-induced asymmetries previously linked to lateralized motor behavior across species and suggest that aspects of left–right brain function in mammals can be ontogenetically influenced by inner ear input. Consistent with inner ear input contributing to motor asymmetry, we also show that, in humans with normal ear function, the motor-dominant hemisphere, measured as handedness, is ipsilateral to the ear with weaker vestibular input.

## Author summary

Despite a long-standing fascination with asymmetries in left–right brain function, very little is known about the causes of functional brain asymmetry in mammals, which appear independent of the mechanisms that create anatomical asymmetries during development. Asymmetries in motor function are a common example and include preferred turning direction, handedness, and footedness. In this study, using mouse models, we establish a causal link between transient imbalances in degenerating inner ear function and the establishment of stable asymmetries in neural pathways that regulate motor activity and in motor behavior. Our study also suggests that shared mechanisms may underlie lateralized motor behaviors across mammalian species. For example, we show that in humans with normal ear function, the strength of the vestibular response from each ear in the forebrain correlates with asymmetric motor behavior, measured as handedness. In a broader sense, our study reveals a conceptually novel role for sensory input in shaping the asymmetric distribution of brain function, a process for which there is otherwise no clear mechanism.

## Introduction

Asymmetries in motor behavior are broadly defined as the uneven use of the right or left sides of the body, which can manifest as asymmetries in hand or foot use, eye movements, head or trunk turning, and in animal tail position or use. Asymmetries can be typical in a population—for example, right-handedness or footedness in humans—or atypical if they deviate from the norm. Why increased incidences of atypical asymmetries in motor behavior are associated with inner ear defects remains unclear. For instance, it is unknown why a 2-fold increase in left- or mixed-handedness is observed in children with congenital and early-acquired postnatal deafness [1–3]. In studies that find an increased incidence of left- or mixed-handedness in deaf children, it is possible that some of these children are also vestibularly impaired because inner ear auditory dysfunction is often accompanied by undiagnosed vestibular dysfunction [4–6]. This leaves open the possibility in these cases of a link between asymmetries in motor behavior and vestibular rather than auditory dysfunction. Consistent with a possible involvement of vestibular input in causing motor asymmetries, monaural vestibular stimulation in normal humans activates cortical and striatal areas more on the side opposite to the dominant hemisphere for handedness; such that in right-handed individuals (i.e., those with a left-dominant hemisphere), higher vestibular activation occurs in the right hemisphere and vice versa for left-handed individuals [7,8]. In patients with bilateral peripheral vestibular dysfunction, this stereotypic asymmetric activation of vestibular cortical and subcortical structures is often disrupted [8]. These studies raise the possibility that vestibular dysfunction can establish a long-lasting atypical asymmetry in brain regions that regulate motor behavior, such as the cortex and striatum.

Models of atypical asymmetries in motor behavior in nonhuman animals are rare. One exception is a turning bias, or directional circling, which can occur across species [9–11]. In humans, based on automated rotometer measures, there is a natural or typical tendency to turn in a preferred direction, which correlates with handedness [12–16]. Atypical turning asymmetries can be linked to neurological and neuropsychiatric disorders such as hemi-Parkinson, schizophrenia, and ischemia, resulting in an exaggerated turning in a preferred direction [11,17–20].

In rodents, a turning bias in one direction due to unilateral 6-hydroxydopamine–induced nigrostriatal lesions and the co-administration of dopaminergic modulators is an established model of an atypical lateralized motor behavior [21–24]. This turning bias is regulated in part by asymmetries in striatal dopamine signaling. Paw preference, the acquired tendency to use mostly the left or right paw during a repeated food-reaching task, also correlates with an asymmetry in dopamine levels in the striatum [25]. Similarly, in human disorders such as hemi-Parkinson or schizophrenia, asymmetric changes in dopamine signaling also appear to underlie atypical turning biases [11,16–20].

Although studies on turning biases in rodents and humans have largely focused on changes in dopamine signaling in the striatum, glutamatergic input from the cortex may also be key. Cortical glutamatergic input converges with dopaminergic input onto the principal projection neurons of the striatum, the gamma-Aminobutyric acid-producing (GABAergic) medium spiny neurons, to regulate motor output, suggesting that input from the cortex may also contribute to asymmetric striatal output and therefore asymmetric motor behavior. Examples of alterations in glutamatergic input causing atypical motor asymmetries include patients with cortical ischemic lesions and exhibiting hemispatial neglect [11,26].

What factors, in the absence of unilateral striatal damage or experimental manipulations, can cause asymmetric motor behavior remain unknown. Similar to circling rodents with severe unilateral 6-hydroxydopamine–induced nigrostriatal lesions [21–24], rodents with congenital inner ear dysfunction often also exhibit circling or repetitive turning. These models of inner ear dysfunction may exhibit an asymmetric behavior without the need for brain lesions and administration of neurotransmitter modulators and may therefore present an opportunity to study how early sensory dysfunction might lead to stable atypical asymmetries within the brain centers that regulate motor output. Such studies may reveal conserved mechanisms that promote lateralized motor behaviors across species and how, in some cases, they may be ontogenetically established by inner ear input.

Here, using mice with congenital inner ear dysfunction, we obtain evidence that transient and uneven inner ear failure can establish atypical asymmetric changes in neurotransmitter components, cortico-striatal physiology, and motor behavior. We also find that, in humans with normal hearing, the strength of the vestibular response in the forebrain induced from each ear correlates with motor asymmetries in handedness, suggesting that the mechanisms underlying inner ear–induced lateralized motor behavior may be conserved across mammalian species and may apply to both atypical and typical motor asymmetries.

## Results

### Ear defects associate with asymmetric motor behavior in mice

Although rodents scarcely exhibit overt asymmetries in motor behavior, mutant mice and rats with bilateral inner ear defects in the cochlea (for hearing) and vestibular labyrinth (for balance) often display a circling or repetitive turning behavior [24]. To establish whether such mutants can serve as a model of atypically acquired asymmetric motor behavior, we first established the extent to which each individual mouse might have a bias in the direction that it turns using several genetic models of inner ear dysfunction: mice with null or conditional mutations in the Na^+^-K^+^-2Cl^−^ cotransporter gene, Solute carrier 12a2 (*Slc12a2)* (null, *Slc12a2*^K842^*^/K842^*, and conditional, Forkhead box g1 [*Foxg1*]^*Cre/+*^;*Slc12a2*^*fx/fx*^) [27,28]; transgenic mice carrying the bacterial artificial chromosome (BAC) 316.23 that overexpress the T-box transcription factor-1 gene (*Tbx1*) associated in humans with hearing loss and mental disorders [29–31]; and mice with a conditional loss of *Tbx1* in tissues that include the inner ear (Paired box 2 [*Pax2*]*-Cre;Tbx1*^*fx/fx*^ [28]) (S1 Fig).

**Fig 1:**
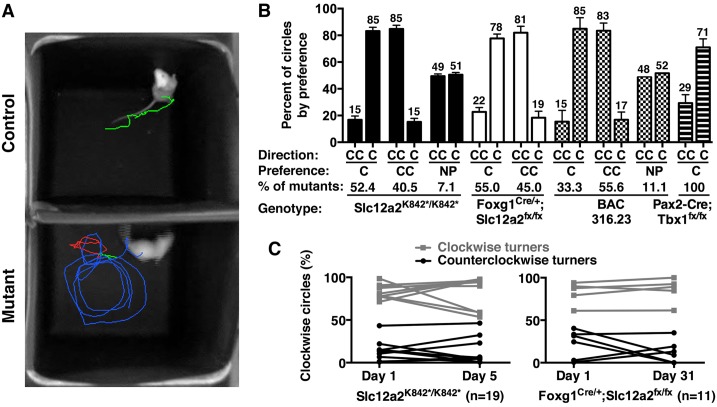
Asymmetric motor behavior in mice is associated with inner ear dysfunction. (A) Thirty-second trace of an *Slc12a2*^K842^*^/K842^* and control mouse. Turns: blue, CC; red, C; green, none. (B) The majority of mutant mice have a bias in the C or CC direction. *Slc12a2*^K842^*^/K842^*, C: *n* = 22; CC: *n* = 17; NP: *n* = 3. *Foxg1*^*Cre/+*^;*Slc12a2*^*fx/fx*^, C: *n* = 11; CC: *n* = 10. BAC 316.23, C: *n* = 3; CC: *n* = 5; NP: *n* = 1. *Pax2-Cre;Tbx1*^*fx/fx*^, C: *n* = 4; mean ± SEM. See also S1 Data. (C) The directional preference is stable over time. Mean percent of clockwise circles: *Slc12a2*^K842^*^/K842^* C turners, day 1 versus 5, *p* > 0.9999; CC turners, day 1 versus 5, *p* > 0.9999; *Foxg1*^*Cre/+*^;*Slc12a2*^*fx/fx*^ C turners, day 1 versus 31, *p* > 0.9999; CC turners, day 1 versus 31, *p* = 0.42. Mean ± SEM, repeated measures ANOVA with Bonferroni multiple comparison test. See also S1 Data. BAC, bacterial artificial chromosome; C, clockwise; CC, counterclockwise; NP, no preference; SEM, standard error of the mean.

Our analysis revealed that, for each mutant genotype, the vast majority of individual 2– to 3–month-old adult mice had a strong bias in their turning direction, either clockwise or counterclockwise (Fig 1A and 1B, S1 Data, and S1 Movie). Littermate controls for each mutant genotype did not circle (S1 Fig; S1 Data). We focused on *Slc12a2* mutants because of their robust phenotype. The preferred turning direction for *Slc12a2*^K842^*^/K842^* and *Foxg1*^*Cre/+*^;*Slc12a2*^*fx/fx*^ mutants was maintained from day 1 of testing to days 5 and 31, respectively (Fig 1C, S1 Data). These data suggest that the majority of *Slc12a2* mutants exhibit a strong tendency to turn in one direction, which is stably maintained well beyond 2 to 3 weeks of age, when inner ear function has effectively failed in both ears [27,28].

### Turning direction is an indicator of asymmetric brain physiology

**Fig 2:**
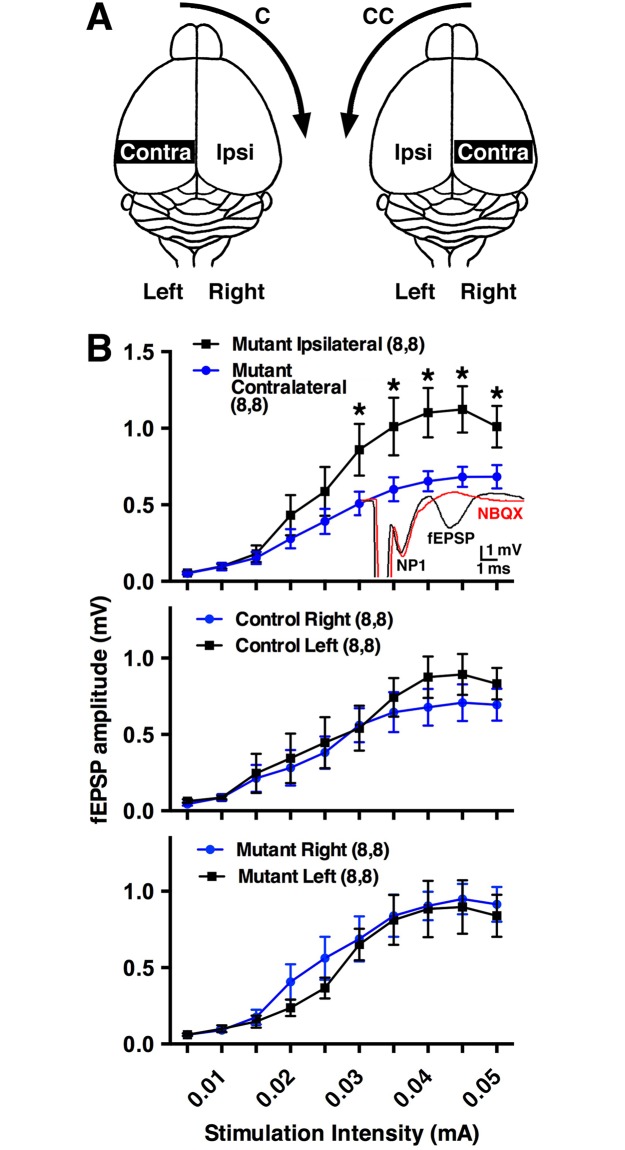
Electrophysiological asymmetry in the cerebral hemispheres correlates with the preferred turning direction. (A) Illustration of the contralateral and ipsilateral hemispheres regarding turning direction. (B) fEPSPs were greater (*p* = 0.017) on the ipsilateral compared to contralateral side in *Slc12a2*^K842^*^/K842^* mice. Inset: standard cortico-striatal fEPSP recording (black) and the sensitivity of the EPSP component to NBQX, an AMPA receptor antagonist (red); NP1: presynaptic fiber volley. fEPSPs between left and right hemispheres of littermate controls (*p* = 0.94) or of mutants (*p* = 0.96). Numbers in parentheses: number of recordings, number of mice. Mean ± SEM, two-way repeated measures ANOVA. See also S1 Data. AMPA, α-amino-3-hydroxy-5-methyl-4-isoxazolepropionic acid; C, clockwise; CC, counterclockwise; EPSP, excitatory postsynaptic potential; fEPSP, field excitatory postsynaptic potential; NP1, negative peak 1; SEM, standard error of the mean.

Strong directional turning biases in rodents and humans can result from experimental lesions or ischemia, respectively, that alter the pathways in the forebrain related to motor function [11,24]. A stably maintained asymmetric motor activity in our mouse models should also be reflected by asymmetric activity in the brain. We examined the cortico-striatal neural pathway, which regulates motor activity, by determining the relative levels of cortico-striatal synaptic transmission in each hemisphere of adult *Slc12a2*^K842^*^/K842^* mutants and littermate controls. Extracellular field recordings performed on acute sagittal brain slices showed that field excitatory postsynaptic potentials (fEPSPs) were significantly greater on the side ipsilateral to the preferred direction of circling in mutant mice when compared to the contralateral side (Fig 2B, S1 Data). As controls, no difference was observed in the amplitude of the fEPSPs for pooled values between the left or right hemispheres of littermate controls or mutants (Fig 2B), the latter of which should not show a difference given that individual mutants exhibit a turning preference independent of their left and right sides. No differences were detected in paired-pulse ratios, afferent volley amplitude, and the slope of the input-output curves normalized to the peak response between hemispheres in all comparisons (S2 Fig, S1 Data). These data are consistent with a postsynaptic difference in cortico-striatal synaptic transmission underlying the observed difference in the amplitude of the cortico-striatal fEPSP between the contralateral and ipsilateral hemispheres of *Slc12a2*^K842^*^/K842^* mice.

**Fig 3:**
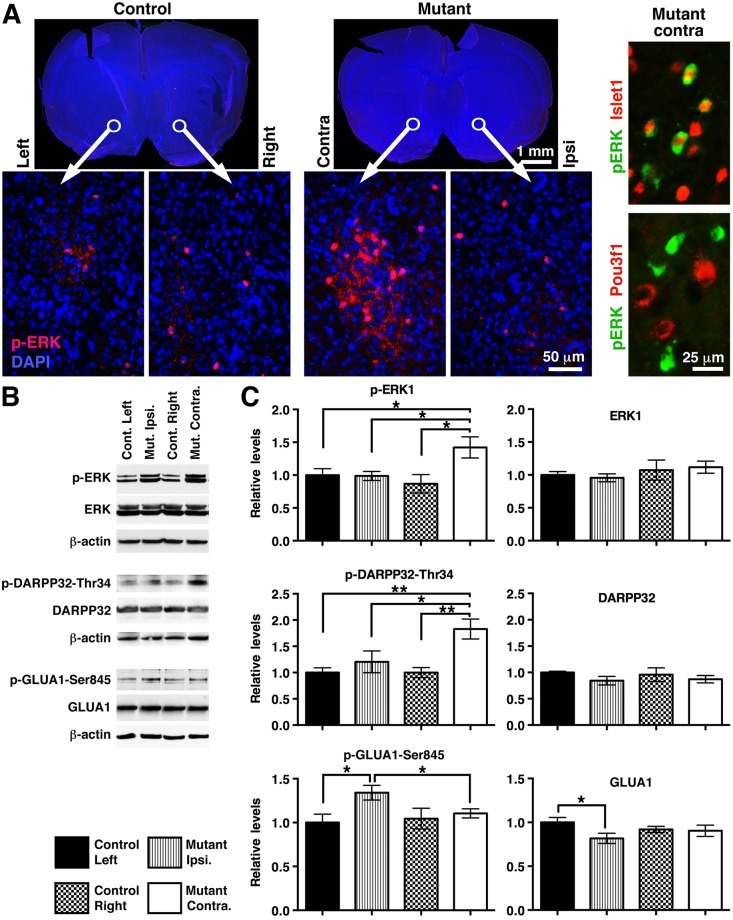
Molecular asymmetries in striatal hemispheres correlate with preferred turning direction in *Slc12a2*^K842^*^/K842^* mice. (A) Left panels: p-ERK immunostaining within the nucleus accumbens. Right panels: costaining of the mutant ventral striatum on the contralateral side with anti–p-ERK and anti-Islet1 or anti–p-ERK and anti-Pou3f1. (B) Western blots of striatal protein. β-actin, loading control. (C) Western blot quantification. Values are normalized to β-actin. * = *p* < 0.05; ** = *p* < 0.005. Mean ± SEM, two-tailed student *t* test; *n* = 6 mice per genotype. See also S1 Data. p-ERK, phosphorylated extracellular signal-regulated kinase; SEM, standard error of the mean.

The electrophysiological asymmetry observed between contra- and ipsilateral hemispheres of *Slc12a2*^K842^*^/K842^* mutants should translate to asymmetric levels of certain proteins involved in neurotransmission. Consistent with this possibility, clusters of cells strongly stained for phosphorylated extracellular signal-regulated kinase (p-ERK), an integrator of glutamate and dopamine signaling [32–35], were detected in the contralateral but not ipsilateral nucleus accumbens of mutants but undetected in either side of littermate controls (Fig 3A). Co-immunostaining for p-ERK with Islet1 (a marker for Dopamine type 1 receptor [D1R]-expressing neurons [36]) or Pou3f1 (a marker for D2R-expressing neurons [36]; Fig 3A) revealed that 68.1% of p-ERK^+^ cells were also Islet1^+^ (234/343, *n* = 6 mice), whereas 0.4% of p-ERK^+^ cells were also Pou3f1^+^ (1/231, *n* = 6 mice), suggesting that signaling through D1R- but not D2R-expressing neurons is particularly affected in the mutants.

Western blot analysis of striatal lysates done for 24 proteins related to neurotransmitter signaling revealed 3 that were atypically distributed between striatal hemispheres in *Slc12a2*^K842^*^/K842^* mutants: DARPP-32 phosphorylated at threonine 34 (p-DARPP32-Thr34) and p-ERK1 were elevated in the contralateral hemisphere, and Glutamate AMPA-Receptor 1 phosphorylated at serine 845 (p-GLUA1-Ser845) was elevated in the ipsilateral hemisphere (Fig 3B and 3C, S1 Data, and S3 Fig). These differences are consistent with a relative increase in fEPSPs and glutamate signaling through GLUA1 in the ipsilateral striatal hemisphere leading to a relative decrease in DARPP32 phosphorylation on Thr34, resulting in extracellular signal-regulated kinase (ERK) deactivation [32,33]. Altogether, our results indicate that interhemispheric molecular and electrophysiological differences correlate with the preferred turning direction, a behavior that can be used as an exemplar for motor asymmetry.

### Asymmetric turning behavior is regulated by striatal p-ERK levels

**Fig 4:**
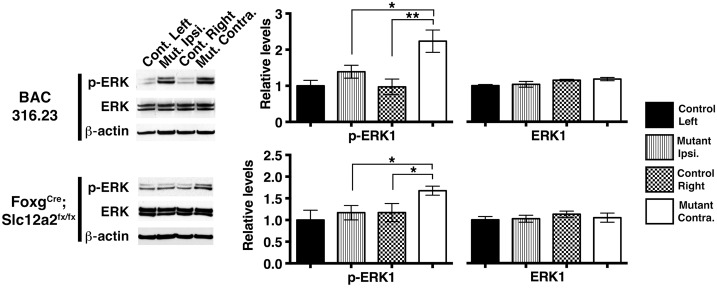
The association between asymmetric striatal p-ERK and the preferred turning direction extends to multiple mouse models of inner ear dysfunction. Western blot analysis and quantification indicate that ERK phosphorylation is up-regulated in the striatal hemisphere contralateral to the preferred direction of signaling in *Foxg1*^*Cre/+*^;*Slc12a2*^*fx/fx*^ (contralateral versus ipsilateral, *p* = 0.027, *n* = 6 for mutants and *n* = 6 for controls) and BAC 316.23 mutants (contralateral versus ipsilateral, *p* = 0.045, *n* = 5 for mutants and *n* = 3 for controls). No significant differences in unphosphorylated ERK were detected between hemispheres in all comparisons. Mean ± SEM, two-tailed t-test. See also S1 Data. BAC, bacterial artificial chromosome; ERK, extracellular signal-regulated kinase; p-ERK, phosphorylated extracellular signal-regulated kinase; SEM, standard error of the mean.

**Fig 5:**
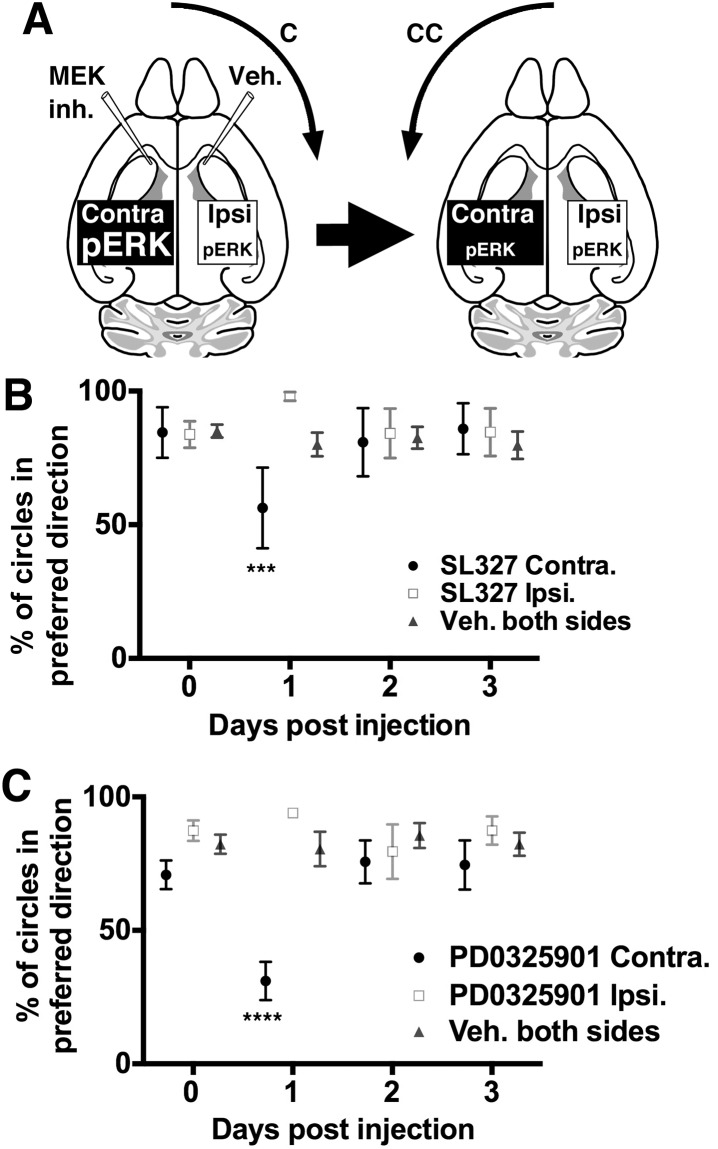
Signaling mediated through striatal ERK contributes to lateralization. (A) Illustration of the hypothesized dependence of the preferred turning direction on asymmetric striatal p-ERK levels and how SL327 inhibition could reduce or reverse the directional preference; C, CC. (B) SLC327-inhibition of p-ERK in the contralateral ventral striatum and vehicle in the ipsilateral side of *Slc12a2*^K842^*^/K842^* mutants reduces clockwise circling 1 day post injection (*n* = 4; *p* = 0.0007 with two-tailed Chi-square and Fisher exact tests), while bilateral vehicle has no effect (*n* = 7). SL327 injected ipsilaterally trended toward increasing the already high clockwise circling 1 day post injection (*n* = 7). Repeated measures ANOVA with the Tukey multiple comparison test; mean ± SEM. See also S1 Data. (C) PD0325901 inhibition of p-ERK in the contralateral ventral striatum and vehicle in the ipsilateral side of *Slc12a2*^K842^*^/K842^* mutants also reduces clockwise circling 1 day post injection (*n* = 8; *p* < 0.0001 with two-tailed Chi-square and Fisher exact tests), while bilateral vehicle has no effect (*n* = 8). PD0325901 injected ipsilaterally, as with SL327, trended toward increasing the already high clockwise circling 1 day post injection (*n* = 8). Repeated measures ANOVA with the Tukey multiple comparison test; mean ± SEM. See also S1 Data. C, clockwise; CC, counterclockwise; ERK, extracellular signal-regulated kinase; MEK, mitogen activated protein kinase kinase; p-ERK, phosphorylated extracellular signal-regulated kinase; SEM, standard error of the mean.

The changes observed in GLUA1 phosphorylation (Fig 3) and cortico-striatal fEPSPs (Fig 2) suggest that glutamate signaling is involved in asymmetry. The changes observed in DARPP32 phosphorylation (Fig 3) along with previous evidence establishing a role for dopamine in asymmetric motor behavior [11,16–25] are consistent with dopamine signaling also playing a role in the mice described here. Dopamine and glutamate signaling cascades in the striatum converge to regulate the phosphorylation of ERK, which promotes stable changes in gene expression leading to long-term adaptations in cellular function and behavior [34,35,37]. p-ERK is up-regulated in the striatal hemisphere contralateral to the preferred turning direction in *Slc12a2*^K842^*^/K842^* mutants (Fig 3) as well as other mutants, including *Foxg1*^*Cre*^;*Slc12a2*^*fx/fx*^, BAC 316.23 mutants (Fig 4, S1 Data), and *Tbx1*^*Cre*^;*Slc12a2*^*fx/fx*^ mutants (see below). If p-ERK asymmetry is required for a predominant turning direction, then pharmacologically inhibiting ERK phosphorylation in the contralateral striatum should minimize or reverse the functional imbalance, reducing or reversing the preference in direction (Fig 5A).

Stereotaxic delivery of the mitogen activated protein kinase kinase (MEK)/ERK kinase inhibitor, SL327, into the ventral striatum results in an approximate 43% decrease in p-ERK (S4A Fig, S1 Data). As hypothesized, adult *Slc12a2*^K842^*^/K842^* mutants injected with SL327 on the contralateral side and vehicle on the ipsilateral side underwent a decrease or reversal of their initial predominant circling direction on day 1 and returned to their baseline turning direction at 2 and 3 days post injection (Fig 5B, S1 Data). These response times appear too rapid to invoke adaptive compensatory mechanisms and are likely directly due to reduced unilateral striatal p-ERK activity. Conversely, *Slc12a2*^K842^*^/K842^* mutants injected with SL327 on the ipsilateral side and vehicle on the contralateral side showed an increase in their predominant circling direction on day 1, but the difference was not statistically significant (Fig 5B). *Slc12a2*^K842^*^/K842^* mutants injected with vehicle on both sides did not undergo a significant change in their predominant circling direction throughout the 3-day observation period (Fig 5B). To confirm these findings, we repeated these experiments using an independent MEK inhibitor, PD325901, also previously shown to reduce p-ERK levels in the striatum [38]. As with SL327, PD325901 injected in the contralateral but not ipsilateral side reduced the preferred circling direction on day 1 post injection (Fig 5C, S1 Data). Together, these findings suggest that directional circling results from the asymmetric activity of factors that regulate levels of p-ERK in the striatum.

### Impaired inner ear function causes atypical brain asymmetry

Thus far, we have shown that asymmetric turning is the result of asymmetric cerebral activity. It remains unclear whether this brain asymmetry is caused by inner ear dysfunction rather than by loss of *Slc12a2* function directly in the brain. To address this question, we conditionally deleted *Slc12a2* from the inner ear using the *Tbx1*^*Cre*^ mouse line to cause deafness and vestibular impairment while leaving *Slc12a2* expression intact in neuronal and glial brain cells.

**Fig 6:**
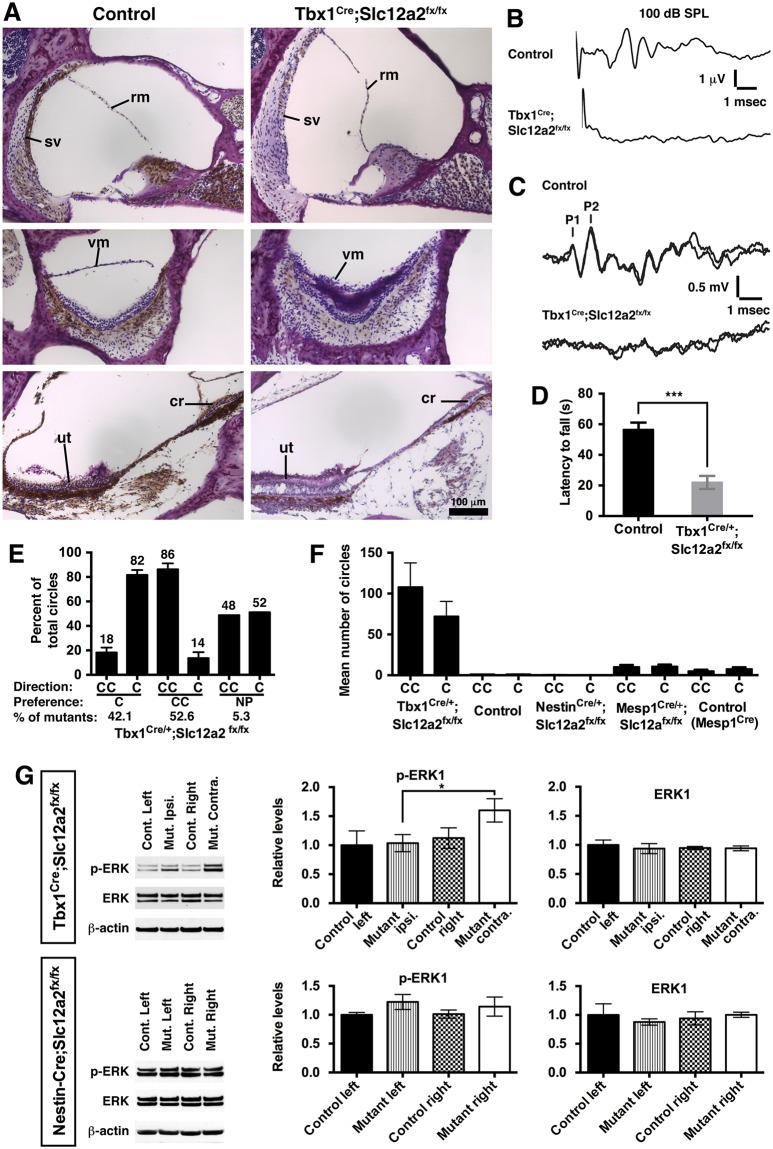
Atypical asymmetries in brain function and behavior are caused by inner ear dysfunction. (A) Immunohistochemical staining of inner ear sections showed reduced SLC12A2 expression (brown) in *Tbx1*^*Cre/+*^;*Slc12a2*^*fx/fx*^ mutants (Nissl counterstain, purple) and defects of the cochlea (top panel) and vestibular structures: saccular vestibular membrane collapse (middle) and ut and cr degeneration (bottom). (B) ABR testing revealed no waveforms at 100 decibels, sound pressure level, in 6-week-old mutants, indicative of deafness. (C) VsEP (P1, first peak; P2, second peak) were absent in mutants, indicative of vestibular impairment. (D) Mutants performed poorly on the rotarod test (*p* < 0.0001), consistent with impaired balance. Mean ± SEM, *n* = 8 mice/genotype, two-tailed *t* test. See also S1 Data. (E) Open field recordings of *Tbx1*^*Cre/+*^;*Slc12a2*^*fx/fx*^ mutants showed a turning bias in either the C (*n* = 8) or CC (*n* = 10) direction (NP, *n* = 1). Mean ± SEM. See also S1 Data. (F) *Tbx1*^*Cre/+*^;*Slc12a2*^*fx/fx*^ mutants circle (*n* = 37), whereas littermate controls (*n* = 10) and *Nestin-Cre;Slc12a2*^*fx/fx*^ mutants do not (*n* = 5). *Mesp1*^*Cre/+*^;*Slc12a2*^*fx/fx*^ mutants, in which Slc12a2 expression is lost from the brain vasculature (Antoine et al., 2017), also do not circle. Mean ± SEM. See also S1 Data. (G) Western blots of striatal lysates from *Tbx1*^*Cre/+*^;*Slc12a2*^*fx/fx*^ mutants showed greater p-ERK1 in the contralateral relative to ipsilateral hemisphere, normalized to β-actin (*p* = 0.046, *n* = 6). Asymmetries in p-ERK1 levels were not detected in *Nestin-Cre;Slc12a2*^*fx/fx*^ mutants (*n* = 3). Mean ± SEM, two-tailed *t* test. See also S1 Data. ABR, auditory brain stem response; C, clockwise; CC, counterclockwise; cr, cristae; NP, no preference; p-ERK, phosphorylated extracellular signal-regulated kinase; rm, Reissner’s membrane; SEM, standard error of the mean; SPL, sound pressure level; sv, stria vascularis; ut, utricle; vm, vestibular membrane; VsEP, vestibular sensory evoked potential.

First, we confirmed that *Tbx1*^*Cre/+*^;*Slc12a2*^*fx/fx*^ mutants display morphological defects of the cochlea and vestibular labyrinth, lack an auditory brain stem response (ABR), are deaf by 6 weeks of age, and lack detectable vestibular function as assessed by vestibular sensory evoked potentials (VsEPs) and performance on a rotarod test (Fig 6A–6D, S1 Data). Fate-mapping studies show that *Tbx1*^*Cre*^ does not drive recombination in neuronal or glial brain cells, but recombination is observed in vascular endothelial cells in the cerebrum [39,40]. However, *Mesp1*^*Cre/+*^;*Slc12a2* mutants, which lack *Slc12a2* expression throughout mesodermal derivatives, including the brain vasculature [39], did not circle (Fig 6F). Therefore, a behavioral phenotype in *Tbx1*^*Cre/+*^;*Slc12a2*^*fx/fx*^ mutants should not be due to loss of *Slc12a2* in the central nervous system (CNS).

As with *Slc12a2*^K842^*^/K842^* mutants, adult *Tbx1*^*Cre/+*^;*Slc12a2*^*fx/fx*^ mutants exhibited asymmetric circling, whereas littermate controls did not circle (Fig 6E and 6F, S1 Data). Therefore, inner ear dysfunction can establish an asymmetric motor behavior. Further supporting this conclusion, adult mice from an independent mutant line, *Pax2-Cre;Tbx1*^*fx/fx*^, in which *Cre* recombines in otic vesicle–derived cells in addition to the mid-hindbrain area, also exhibited asymmetric circling (Fig 1B and S1 Fig). Because *Tbx1* is not expressed in neuronal or glial cells, the behavioral phenotype of *Pax2-Cre;Tbx1*^*fx/fx*^ mutants, like *Tbx1*^*Cre/+*^;*Slc12a2*^*fx/fx*^ mutants, is ascribable to loss of gene expression in otic vesicle derivatives resulting in inner ear defects. In addition, *Nestin-Cre;Slc12a2*^*fx/fx*^ mice, in which gene deletion occurs prior to the onset of both neurogenesis and *Slc12a2* expression and in which SLC12A2 protein is lost from the entire CNS, do not circle (Fig 6F) [28]. Together, these mutants indicate that inner ear dysfunction alone can result in a persistent asymmetric behavior.

To determine whether inner ear dysfunction also causes asymmetric changes in the striatum, levels of p-ERK were examined. Western blot analysis of striatal lysates of *Tbx1*^*Cre/+*^;*Slc12a2*^*fx/fx*^ mutants revealed greater levels of p-ERK1 in the contralateral relative to the ipsilateral hemisphere, similar to that observed for *Slc12a2*^K842^*^/K842^*, *Foxg1*^*Cre/+*^;*Slc12a2*^*fx/fx*^, and BAC 316.23 mutants (Figs 3, 4 and 6G and S1 Data). Asymmetries in p-ERK1 levels were not detected when comparing the hemispheres of littermate controls or *Nestin-Cre;Slc12a2*^*fx/fx*^ mutants, in which SLC12A2 was lost from the whole CNS (Fig 6G). Neither asymmetries in nor changes in absolute levels of total ERK were detectable in all comparisons (Fig 6G). These data suggest that inner ear dysfunction, rather than a CNS defect, induces interhemispheric asymmetry in striatal function, which causes asymmetric motor behavior.

### An early imbalance in inner ear dysfunction establishes asymmetry

Inner ear dysfunction is sufficient to establish asymmetric motor function in the brain. However, mice that have undergone complete surgical removal of both inner ears at the same time do not display a predominant circling direction [41]. In contrast, asymmetric circling has been observed with progressive ear degeneration in postnatal animals given the ototoxic drug streptomycin [42]. Therefore, we hypothesized that one hemisphere is established as motor dominant as a result of a transient left–right imbalance in degenerating ear function.

Consistent with this possibility, functional inner ear asymmetries are detected in our circling mouse models. BAC 316.23 mutants have differences in the degree of hearing loss between the left and right ear [43]. *Tbx1*^*Cre/+*^;*Slc12a2*^*fx/fx*^ mutants prior to 4 weeks of age exhibit a difference between right and left ears in their ABR response threshold (5.7 ± 2.9 dB difference from the average threshold of 76 ± 6 dB; *n* = 6) before becoming equally profoundly hearing impaired in both ears by 4 weeks of age (Fig 6B) [39]. And although obtaining ear specific vestibular evoked responses in mice is difficult [44], *Tbx1*^*Cre/+*^;*Slc12a2*^*fx/fx*^ mutants exhibit a difference in the severity of collapse of the vestibular membrane of the saccula, a measure of vestibular impairment [27], between the left and right ear at 5 to 6 weeks of age, just prior to complete vestibular failure (S5 Fig) [39].

**Fig 7:**
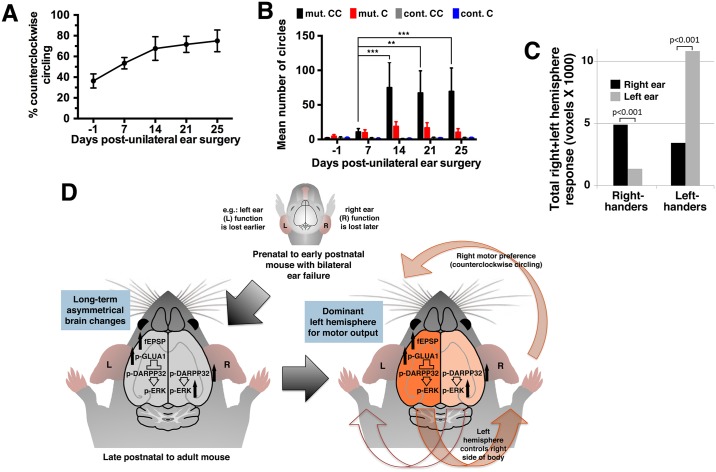
Imbalanced inner ear dysfunction determines the preferred turning direction. (A) Open field recordings of *Tbx1*^*Cre/+*^;*Slc12a2*^*fx/fx*^ mutants (*n* = 10) before (day −1) and after unilateral surgery on the left ear showed a preference for CC turning by 14 days. See also S1 Data. (B) Average CC and C turns for the mutants in panel A and littermate controls (*n* = 10) with unilateral ear surgery. Two-way RM ANOVA with Tukey’s multiple comparison test (between mutant CC and C: at day 14, *p* = 0.0051; day 21, *p* = 0.0019; day 25, *p* < 0.0001). See also S1 Data. (C) Activation during caloric vestibular stimulation of the right and left ear in a right- and left-hander with numbers of voxels exhibiting significant differences in effect size between left and right hemispheres (paired *t* test; *n* = 12 right-handers, *n* = 12 left-handers; *p* < = 0.001). See also S1 Data. (D) Model of ear-induced lateralization. C, clockwise; CC, counterclockwise; fEPSP, field excitatory postsynaptic potential; p-ERK, phosphorylated extracellular signal-regulated kinase; RM, repeated measure.

Unlike other mutants examined in this study that display complete loss of inner ear function and onset of circling upon becoming ambulant at 2 to 3 weeks of age, *Tbx1*^*Cre*^-driven *Slc12a2* mutants, although exhibiting profound hearing loss by 4 weeks of age, only exhibit loss of vestibular function and circling after 5 weeks of age [39]. This extended time to reach vestibular failure in *Tbx1*^*Cre*^;*Slc12a2* mutants not only suggests that vestibular rather than auditory dysfunction underlies circling [39] but also provides an opportunity to manipulate inner ear function before the onset of lateralized turning. To test the hypothesis that a persistent turning direction is the result of an early transient imbalance in inner ear dysfunction, we surgically delivered ototoxic drugs into 1 inner ear of *Tbx1*^*Cre/+*^;*Slc12a2*^*fx/fx*^ mutants and controls at 3 to 4 weeks of age before complete inner ear failure and the onset of asymmetric turning. Prior to surgery, *Tbx1*^*Cre/+*^;*Slc12a2*^*fx/fx*^ mice, like all mice, occasionally turned 360° in either direction, but no preference was detected in their turning direction (S1 Table). At 7, 14, 21, and 25 days after surgery to the left inner ear, control mice did not circle, although they exhibited the expected head tilting towards the lesioned ear. Mutants, however, developed a strong preference to turn counterclockwise (Fig 7A and 7B, S1 Data), indicating greater use of the right limbs (contralateral to the weaker ear) and a dominant output from the left hemisphere (ipsilateral to the weaker ear). Altogether, these data suggest that asymmetric motor activity can be due to an early asymmetric imbalance of the inner ears working through the basal ganglia to control movement. Importantly, the preferred circling direction in mice persists long after function of both ears is undetectable, indicating that the cerebral asymmetry is stably maintained.

### Asymmetric vestibular input in humans correlates with handedness

We previously reported that handedness and vestibular dominance are located in opposite hemispheres [7]. We speculated that the vestibular hemispheric dominance, which matures early during ontogenesis, could determine handedness. In light of the current findings, we recalculated our data from Dieterich et al. [7] to test whether the strength of the vestibular input to the brain is itself asymmetric and also correlates with handedness. Consistent with our expectations, in individuals with normal ear function subjected to vestibular stimulation by caloric irrigation followed by positron emission tomography (PET) scan imaging of blood flow changes in the brain, we found that the total cortical response, a measure of the strength of vestibular input, is asymmetric and correlates with handedness. A higher response was observed to left ear stimulation in left-handers and right ear stimulation in right-handers (Fig 7C). Hence, the weaker ear is ipsilateral to the motor-dominant hemisphere (right hemisphere in left-handers and left hemisphere in right-handers). This observation, along with our previous studies [7], suggests that uneven inner ear input may contribute to brain asymmetry for motor functions, including handedness, across mammalian species.

## Discussion

The present report has identified a role for an early inner ear imbalance in determining the dominant hemisphere for an atypical and asymmetric motor behavior (Fig 7D). These findings suggest that atypical (and perhaps normal) functional motor asymmetries in mammals can be a product of sensory input rather than simply an innate program of brain development. Our findings also suggest that the asymmetric circling behavior induced by inner ear dysfunction is maintained over the long term. Interestingly, caloric stimulation has also been found to asymmetrically impact behavior in the short term. For example, unilateral cold-water vestibular stimulation can, in the short term, improve memory based specifically in the hemisphere contralateral to the stimulated ear (word recall for the left hemisphere and recall of object location for the right hemisphere) [45]. However, the mechanism underlying such ear-induced short-term effects may differ from those that underlie long-term motor asymmetries.

Our findings also suggest that the motor-dominant hemisphere has higher levels of glutamate neurotransmission and perhaps lower levels of dopamine signaling (the latter being inferred from the lower striatal levels of p-DARPP32-Thr34 and ERK activity). Therefore, the *Slc12a2*^K842^*^/K842^* mice described here are similar to rats with unilateral lesions of the nigro-striatal tract that are administered modulators of dopamine signaling: both *Slc12a2*^K842^*^/K842^* mice and lesioned rats circle predominantly toward the dominant motor hemisphere, which exhibits lower dopamine signaling [24]. Likewise, in rats and mice that exhibit a preference in paw usage, lower levels of dopamine are also observed in the dominant striatal hemisphere (i.e., contralateral to the preferred paw) [25,46]. Therefore, uneven striatal levels of glutamate and dopamine likely underlie aspects of atypical and perhaps normal asymmetric motor behaviors from rodents to humans [11,14,15,19].

One possibility for how ear imbalances might establish atypical asymmetries for motor function is through vestibular-regulated development of spatial attention. In this case, the dominant motor output of the hemisphere ipsilateral to the more impaired ear would be the indirect result of lower vestibular input into this hemisphere. This would result in a vestibular dominance of the contralateral hemisphere, which can cause a stable shift in spatial attention and orientation of the animal towards the ipsilateral hemifield (similar to human spatial hemineglect), resulting in a locomotor deviation toward the ipsilateral side and—if strong—to circling in this direction [7,47–49]. Another possibility, which is not mutually exclusive with the above, is that the vestibular system is more directly required for modulating development of motor networks independently of the development of spatial attention.

How inputs from the right and left ears can become uneven remains unknown. With failing inner ear function due to genetic diseases or infections, random differences might cause one ear to deteriorate faster than the other, resulting in unbalanced sensory input to the brain. In the absence of ear-related diseases, it is tempting to speculate that normal inner ear function also plays a role in establishing asymmetries in motor activity. In humans—for whom handedness and language are dominant in the left hemisphere with an approximate 9:1 ratio—auditory input, in addition to vestibular input (Fig 7C), might play a role given that the right ear is often more sound sensitive in neonates (“right-ear advantage”) and less susceptible to age-related hearing loss [50–52]. In mice, for which the frequency of the preferred side of circling or left or right paw usage appear similar (Fig 1A) [53], pups may stochastically experience uneven inner ear activity early in life—for example, due to how they are positioned in the mother’s uterus or how their mother positions them in a litter—after which time asymmetric activity could be self-reinforcing and become stabilized. In sum, the present study suggests that a transient asymmetry in inner ear failure establishes long-lasting asymmetric motor function, providing a potential explanation for how the left and right hemispheres become atypically lateralized in disorders that include inner ear dysfunction and perhaps providing a clue to how our hemispheres normally become lateralized for motor functions.

## Materials and methods

### Ethics statement

The experiments described in this study were approved by the Institutional Animal Care and Use Committee of the Albert Einstein College of Medicine, protocol #20170409, in accordance with AAALAC guidelines and the policies of the federal NIH Office of Laboratory Animal Welfare.

### Mice

Mouse lines have previously been described and are as follows: *Slc12a2*^K842^*, *Slc12a2*^*fx*^, *Tbx1*^*Cre*^, *Foxg1*^*Cre*^, *Pax2-Cre*, *Nestin-Cre* [28], BAC 316.23 [43], RCE [54], *Mesp1*^*Cre*^ [55], and *Tbx1*^*fx*^ [56]. Controls in every case are littermates that either do not carry a Cre allele or are heterozygous for the mutant or floxed allele, except for BAC316.23, for which the littermate controls are wild type.

### Behavior

Behavioral testing was done on mice from 3 weeks to 9 months of age between the hours of 12:00 and 18:00. At least 10 mice of each genotype were tested unless otherwise noted. Mice were acclimatized to the testing room for 2 hours prior to open field recordings in a chamber (25 cm × 25 cm × 20 cm, length, width, height) for a 30-minute period while their locomotor and rotational activity was tracked and recorded using Biobserve’s Viewer 2 Tracking Software with Rotation Viewer Plug-in or with Ethovision 8XT with the Multiple Body Points module. Settings defined a circle as a 360° to 540° turn within 2 seconds. Initial test counts on the number and direction of the circles were validated visually and >99% concordance was obtained between software and observer. Chambers were cleaned with 70% ethanol between sessions. No differences between males and females of the same genotype could be detected; their results were pooled. A mouse was considered to have a directional preference for turning if the ratio of clockwise to counterclockwise turns, or vice versa, exceeded 1 standard error from 50:50, which for these experiments translates to a ratio ≥70:30. To assess the reproducibility in the preference of turning direction, each mouse underwent three 15-minute trials at 1 hour apart, and the score for each mouse was the average of the 3 trials. Note that an effect on the preference of right or left paw usage, a less robust form of motor lateralization, was not assessed due to the difficulty of the mice to rear upright in a paw-preference assay.

### Immunohistochemistry

For brains, mice were perfused transcardially with 4% paraformaldehyde in 0.1M phosphate buffer. Brains were post-fixed, 4°C overnight, sunk in 30% sucrose/PBS, embedded in OCT (Tissue-Tek, Torrance, CA), cryosectioned (20 μm), and mounted on slides. Sections were blocked in 10% goat serum (Jackson Immunoresearch Lab, West Grove, PA) in 0.3% Triton-X 100 for 1 hour and incubated overnight at 4°C with primary antibody in blocking solution. Primary antibodies include mouse NeuN (1:100, Millipore, Burlington, MA), rabbit GFAP (1:500, Dako, Santa Clara, CA), goat SLC12A2 (1:50, Santa Cruz Biotechnology, Dallas, TX), rabbit SLC12A2 (1;100, Proteintech Group, Rosemont, IL), rabbit Laminin (1:200, Millipore), rabbit GFP (1:100, Life Technologies, Carlsbad, CA), rabbit p-ERK (1:200, Cell Signaling, Danvers, MA), mouse Islet1 (1:2, Developmental Studies Hybridoma Bank, Iowa City, IA), and mouse Pou3f1/Oct6 (1:50, Millipore). Tissues were washed 5 times in PBS and incubated for 1 hour at room temperature with the relevant secondary antibodies in blocking solution: Alexa Fluor-488 or Alexa Fluor-568-conjugated (1:350, Life Technologies) or horseradish peroxidase-conjugated (1:250, Jackson Immunoresearch). Sections were counterstained with Hoechst 33342 or DAPI, mounted in Fluoromount G (Southern Biotech, Birmingham, AL), and analyzed via conventional fluorescence microscopy (Zeiss AxioSkop2 p). For ears, tissue was fixed in 4% PFA/PBS for 3 days, decalcified in 0.5 M EDTA/PBS at 4°C for 2 to 3 days, paraffin embedded, sectioned (5 μm), deparaffinized with Xylene and ethanol, and rehydrated in distilled water. Blocking and antibody application were as above except the reaction product was visualized with a diaminobenzidine (DAB) peroxidase substrate (Vector Laboratories, Burlingame, CA), counterstained with 1% Cresyl Violet (Sigma, St. Louis, MO), and cover-slipped with Permount (Fisher Scientific, Waltham, MA).

### Cortico-striatal electrophysiology

Extracellular field recordings were performed on acute parasagittal brain slices by positioning a field-recording electrode in the dorso-lateral striatum and a stimulating electrode in the corpus callosum. Whole brains were cut in half sagittally, placed in ice-cold cutting solution ([in mM] 234 sucrose, 2.5 KCl, 1.25 NaH_2_PO_4_, 10.0 MgSO_4_, 1 CaCl_2_, 26 NaHCO_3_, 20 glucose, saturated with 95% O2 and 5% CO2) 5 minutes, sliced (300 μm) in a 50:50 mix of cutting solution (above) and normal external recording solution ([in mM]: 124 NaCl, 2.5 KCl, 1 NaH_2_PO_4_, 26 NaHCO_3_, 1.3 MgSO_4_, 2.5 CaCl_2_,10 glucose), and allowed to recover for >1 hour prior to recording. Solutions were continuously equilibrated with 95% O_2_ and 5% CO_2_ (pH 7.4) and perfused at a flow rate of 2 ml/minute at 30°C to 32 °C. Current (0–0.1 mA) was delivered to the stimulating electrode (bipolar tungsten, MX21XEP, Frederick Haer, Bowdoin, ME) using a stimulus isolator (Iso-flex; A. M. P. I.). Recordings were performed with picrotoxin to inhibit interference from intra-striatal GABAergic synaptic transmission. Three distinct components were observed in all recordings: the stimulation artifact, negative peak 1 (NP1) and NP2 (fEPSP). NP1 was identified as the action potential–derived fiber volley based on its latency, sensitivity to tetrodotoxin (a voltage-gated sodium channel antagonist, 0.001 mM; Tocris) and resistance to NBQX (an AMPA receptor blocker, 0.05 mM), AP5 (an NMDA receptor blocker, 0.1 mM), and picrotoxin (0.1 mM, GABA_A_ receptor antagonist, Tocris). NP2 was not due to direct activation of the striatal neurons by the stimulating electrode current as it displayed sensitivity to tetrodotoxin, while its sensitivity to NBQX and latency confirmed this peak as the fEPSP. Amplitude and slope for each time point were obtained from the average of 5 responses. Paired-pulse responses were evoked at the stimulation intensity that produced the maximal fEPSP. The paired-pulse ratio was defined as the ratio of the amplitude of the second to the first fEPSP elicited by pairs of stimuli at interstimulus intervals of 10, 20, 50, 100, and 200 milliseconds. Data were acquired with a Multiclamp700B amplifier (Axon Instruments, San Jose, CA) and analyzed with Igor Pro 4.09A software (Wavemetrics, Inc., Lake Oswego, OR) offline blind to genotype and left or right hemisphere. Statistical analysis was performed using two-way repeated measures ANOVA at the *p* < 0.05 significance level and reported as mean ± standard error of the mean (SEM).

### Western blotting

Striatal tissue was dissected and homogenized in cold 25 mM Tris-HCl (pH 7.4), 150 mM NaCl, 5 mM EDTA (pH 8.0), 5 mM EGTA (pH 8.0), 0.5% TritonX-100, 1% proteinase and phosphatase inhibitors (Sigma Aldrich), 0.05% SDS, and 0.05% CHAPS. Protein concentration was determined using the BCA kit (Pierce Biotechnology, Inc., Waltham, MA). Protein lysate (30 ug) was resolved on a 4% to 12% Bis-Tris acrylamide gel, transferred to nitrocellulose, blocked for 1 hour, room temperature, in 5% BSA in TBST (0.05% Tween 20), and incubated overnight with primary antibody: GLUA1 (Millipore), GLUA2 (Millipore), vGLUT1 (NeuroMab, Davis, CA), vGLUT2 (NeuroMab), GLUN1 (Millipore), GLUN2A (NeuroMab), GLUN2B (NeuroMab), dopamine receptor 2 (Santa Cruz Biotechnology), tyrosine hydroxylase (Sigma Aldrich), dopamine transporter (Millipore), cannabinoid receptor CB-1 (Thermo Scientific, Waltham, MA), ChAT (Millipore), GIRK/Kir3 (Alomone Labs, Jerusalem, Israel), phospho-ERK1/2-Thr202/Tyr204 (Cell Signaling), ERK1/2 (Cell Signaling), DARPP-32 (Santa Cruz Biotechnology), phospho-DARPP-32-Thr34 (Cell Signaling), phospho-DARPP-32-Thr75 (Cell Signaling), cFos (Santa Cruz Biotechnology), and β-Actin (Sigma Aldrich). Blots were then incubated in a 5% BSA/TBST solution containing the relevant horseradish peroxidase-conjugated secondary antibodies (1:250, Jackson Immunoresearch) for 1 hour at room temperature. Chemiluminescent signals were detected with ECL Plus Western Blotting Detection Reagents (Amercham, Little Chalfont, UK), and band intensities were quantified with ImageJ (NIH). Band densities were normalized to β-Actin, and quantities presented relative to the left-control hemisphere. Significance was assessed with the student two-tailed *t* test.

### Stereotaxic MEK inhibitor injections

Mice were anesthetized with isoflurane and stereotaxically injected in the nucleus accumbens with 2 μl of a 10 μM solution of PD0325901 (in 5% Tween-80 in saline with 50 μg/μl methylene blue) or a 4.7 μg/μl solution of SL327 (in 1XPBS, 50% DMSO, 50 μg/μl methylene blue) or the respective vehicle solution at 0.1 μl/minute at these Bregma coordinates: AP, 1.2 mm; ML, 1.2 mm; DV, 4.2 mm; as described [28]. The needle was left in place for 5 minutes after injection before withdrawal. Mice were injected bilaterally, one side with SL327 and one with vehicle, or both sides with vehicle. Upon completion of behavioral testing, the accuracy of the injection site was confirmed by Nissl counterstaining in perfused tissue and by methylene blue staining in unperfused tissue.

### Vestibular and auditory testing

Mice used for VsEP testing were anesthetized with ketamine:xylazine (18:2 mg/ml) and fitted with subcutaneous electrodes at the back of the skull (nuchal crest, noninverting), behind the pinna (inverting), and at the hip (ground). The heads were secured with a noninvasive clip to a mechanical shaker to receive kinematic stimulation with linear jerk stimuli with 2 millisecond duration presented at 17 pulses/second in both up and down directions relative to the naso-occipital axis (National Instruments modules and custom software were used to deliver stimuli and record responses). Averaged waveforms were derived from 256 primary responses (electrophysiological activity was amplified 200,000 times, filtered at 100–3,000 Hz, and digitized at 10 microseconds/point) and replicated. Hearing was evaluated by testing for ABRs, as reported [28]: auditory-evoked potentials elicited by click stimuli at increasing sound pressure levels from 30 dB to 100 dB at 6 dB increments were recorded.

### Ear surgeries

The surgeries involved making an incision from the base of the concha (external side) to halfway around the external auditory meatus to access the oval window. The tympanum, malleus, and incus were removed through the meatus, the anterior and posterior crura of the stapes were broken, and the stapes footplate removed. Through the oval window, 15 μl of 100 mg/ml neomycin and 200 mg/ml streptomycin were injected into the cochlea and semicircular canals. The middle ear cavity was packed with gelfoam and the external ear sutured.

### Vestibular stimulation by caloric irrigation

Human subjects are those described in Dieterich et al. [7], and caloric irrigation and imaging were as described in that report. Briefly, 24 individuals without histories of otoneurological dysfunction participated in the study: 12 right-handed individuals (30–61 years old, mean 42.4 years, 3 women, 9 men) and 12 strongly left-handed individuals (9–43 years old, mean 27.6 years, all male). The laterality quotient for handedness was determined according to the Edinburgh test [57,58] and was +100 for 10, +80 for 1, and +60 for the other right-hander; −100 in 10 and −80 in 2 of the left-handers. In accordance with the Declaration of Helsinki, all subjects gave their informed written consent to participate in the study, after the experimental procedure and radiation risks had been explained. Experiments were approved by the Ethics Committee of the Technical University Munich and the Radiation Safety Authorities. The right or left ear canals were irrigated with warm water (100 ml, 44°C, 50 seconds), and the horizontal DC electrooculogram were calibrated and recorded as described [7]. In the control condition, individuals lay at rest in the supine position with eyes closed without stimulation. PET scanning and data acquisition were done using a Siemens 951 R/31 PET scanner (CTI, Knoxville, TN) and a total axial field of view of 10.5 cm with no interplane dead space covering the vertex to the upper cerebellum. Corrections and sampling were as described. Regional cerebral blood flow was measured with 10.0 mCi ^15^O-labelled water. The influence of nociceptive, somatosensory, and acoustic sensations was avoided by scanning 25 seconds after irrigation ended when nystagmus was still prominent but sensory sensations had disappeared. Ten-minute intervals between scans allowed for radioactivity decay. Image analysis was performed as described with corrections for randoms, dead time, scatter, and head movements [7].

## Supporting information

S1 Table Example of directional preferences for a *Tbx1*^*Cre/+*^;*Sls12a2*^*fx/f*^ mutant that turns clockwise and another that turns counterclockwise. (DOCX)

S1 Data Excel spreadsheet containing, in separate sheets, the underlying numerical data for all figures. (XLSX)

S1 Movie Simultaneous recording of an *Slc12a2*^K842^*^/K842^* mutant (left chamber) and a littermate control (right chamber). (MP4)

S1 Fig Several mutant mouse lines that circle exhibit cochlear and vestibular inner ear defects. (A) Automated quantification of spontaneous circling behavior during open field locomotor activity confirms that several mutant mouse lines (M) with genetically caused inner ear defects circle, whereas littermate controls (C) do not. *Slc12a2*^K842^*^/K842^* and controls, *n* = 49, 20; *Foxg1*^*Cre/+*^;*Slc12a2*^*fx/fx*^ and controls, *n* = 23, 15; BAC 316.23 and controls, *n* = 10, 6; *Pax2-Cre;Tbx1*^*fx/fx*^ and controls, *n* = 4, 6. Mean ± SEM. (B) Immunohistochemical staining for SLC12A2 (Brown; except for BAC 316.23) of inner ear sections reveal morphological defects: rm collapse in the cochlea, vm collapse in the saccula, and degeneration of the utricle and cristae in *Foxg1*^*Cre/+*^;*Slc12a2*^*fx/fx*^,*Slc12a2*^K842^*^/K842^* and BAC 316.23 mutants. Nissl counterstain (purple). BAC, bacterial artificial chromosome; C, cristae; rm, Reissner’s membrane; SEM, standard error of the mean; U, utricle; vm, vestibular membrane.
(TIF)

S2 Fig No significant asymmetries were detected in presynaptic function or axonal excitability between the cerebral hemispheres of *Slc12a2*^K842^*^/K842^* mutants. (A) No differences were detected in presynaptic function as indicated by paired-pulse ratios (slope of fEPSP no. 2/slope fEPSP no. 1) at 0.05 mA stimulation intensity when comparing the contralateral and ipsilateral hemispheres of *Slc12a2*^K842^*^/K842^*mutants (*p* = 0.67), the left or right hemispheres of littermate controls (*p* = 0.52), or the left or right hemispheres of *Slc12a2*^K842^*^/K842^* mutants (*p* = 0.58). (B) As a measure of neuronal excitability, the amplitude of the action potential component (NP1) of the fEPSP across the range of stimulation intensities was similar between hemispheres in all comparisons. Inset illustrates the presynaptic fiber volley (NP1; black trace) and its sensitivity to tetrodotoxin, a voltage-gated sodium channel inhibitor (red trace) with the EPSP previously blocked with NBQX and AP5. Mean ± SEM, two-way repeated measures ANOVA. (C) No significant difference in the slope of the input-output curves normalized to the peak response was detected between hemispheres in all comparisons. Numbers in parentheses indicate the total number of recordings followed by the number of mice. EPSP, excitatory postsynaptic potential; fEPSP; field excitatory postsynaptic potential; NP1, negative peak 1; SEM, standard error of the mean.
(TIF)

S3 Fig Candidate striatal proteins whose levels were not asymmetrically distributed between hemispheres. Quantification of western blots on striatal lysates from *Slc12a2*^K842^*^/K842^* mutants and littermate controls. Values are normalized to β-actin. *n* = 6 mice/genotype (except *n* = 3 for p-GLUN1-Ser897). Two-tailed unpaired *t* test. For p-ERK2 control right vs mutant contralateral, *p* = 0.0039, and control right versus mutant ipsilateral *p* = 0.0044. CB1, cannabinoid receptor type 1; CHAT, choline acetyltransferase; D2R, dopamine receptor-2; DAT, dopamine transporter; GIRK2, G protein-coupled inwardly-rectifying potassium channel-2; GLUA2, α-amino-3-hydroxy-5-methyl-4-isoxazolepropionic acid receptor (AMPAR) subunit; GLUN1,GLUN2A,GLUN2B, *N*-methyl-D-aspartate receptor (NMDAR) subunits; p-ERK, phosphorylated extracellular signal-regulated kinase; TH, tyrosine hydroxylase; vGLUT1,vGLUT2, vesicular glutamate transporter-1 and 2.
(TIF)

S4 Fig SL327 administered to the striatum decreases the levels of p-ERK. (A) Western blot analysis of striatal lysates from*Slc12a2*^K842^*^/K842^* mutants receiving the MEK inhibitory SL327 or vehicle and controls receiving vehicle. (B) As expected, mutant mice treated with vehicle (*n* = 6) had elevated levels of p-ERK compared to controls treated with vehicle (*n* = 4) (*p* = 0.038). However, levels of p-ERK are reduced in mutants receiving SL327 (*n* = 10) compared with mutants receiving vehicle (*p* = 0.038), such that the levels in mutants receiving SL327 approach those found in control mice treated with vehicle (*p* = 0.95). Mean ± SEM, one-way ANOVA with Tukey’s multiple comparisons test. (C) Levels of unphosphorylated ERK were not affected by SL327 administration. ERK, extracellular signal-regulated kinase; MEK, mitogen activated protein kinase kinase; p-ERK, phosphorylated extracellular signal-regulated kinase; SEM, standard error of the mean.
(TIF)

S5 Fig Uneven collapse of the vestibular membrane in mice correlates with lateralized behavior. Nissl stained sections through the saccula shows uneven levels of vm collapse between the contralateral and ipsilateral ear of *Tbx1*^*Cre/+*^;*Slc12a2*^*fx/fx*^ mutants (*n* = 5), prior to complete vestibular failure. vm, vestibular membrane.
(TIF)

## Abbreviations

ABR: auditory brain stem response
AMPA: α-amino-3-hydroxy-5-methyl-4-isoxazolepropionic acid
BAC: bacterial artificial chromosome
CNS: central nervous system
cr: cristae
D1R: dopamine type 1 receptor
DAB: diaminobenzidine
EPSP: excitatory postsynaptic potential
ERK: extracellular signal-regulated kinase
fEPSP: field excitatory postsynaptic potential
*Foxg1*: Forkhead box g1 gene
GABA: gamma-Aminobutyric acid
MEK: mitogen activated protein kinase kinase
NP1: negative peak 1
*Pax2*: Paired box 2 gene
p-DARPP32-Thr34: DARPP-32 phosphorylated at threonine 34
p-ERK: phosphorylated extracellular signal-regulated kinase
PET: positron emission tomography
p-GLUA1-Ser845: Glutamate AMPA-Receptor 1 phosphorylated at serine 845
rm: Reissner’s membrane
SEM: standard error of the mean
*Slc12a2*: Solute carrier 12a2 gene
sv: stria vascularis
*Tbx1*: T-box transcription factor-1 gene
ut: utricle
VsEP: vestibular sensory evoked potential
