# Supplementary material for: Early uneven ear input induces long-lasting differences in left–right motor function
Source: PLoS Biol. 2018 Mar 13;16(3):e2002988. doi: 10.1371/journal.pbio.2002988 (PMC5849283; doi:10.1371/journal.pbio.2002988)
Supplement: S1 Table — (DOCX) [file pbio.2002988.s001.docx]

| **Age (weeks)** | **Number of circles** | | | |
| --- | --- | --- | --- | --- |
|  | **Mouse 1** | | **Mouse 2** | |
|  | **CC** | **C** | **CC** | **C** |
| **3** | 3 | 4 | 0 | 1 |
| **4** | 7 | 2 | 1 | 3 |
| **5** | 7 | 5 | 2 | 2 |
| **6** | 58 | 102 | 137 | 20 |
| **8** | 16 | 129 | 483 | 46 |
| **23** | 141 | 423 | 40 | 23 |
