# Supplementary figures and images for: Early uneven ear input induces long-lasting differences in left–right motor function
Source: PLoS Biol. 2018 Mar 13;16(3):e2002988. doi: 10.1371/journal.pbio.2002988 (PMC5849283; doi:10.1371/journal.pbio.2002988)

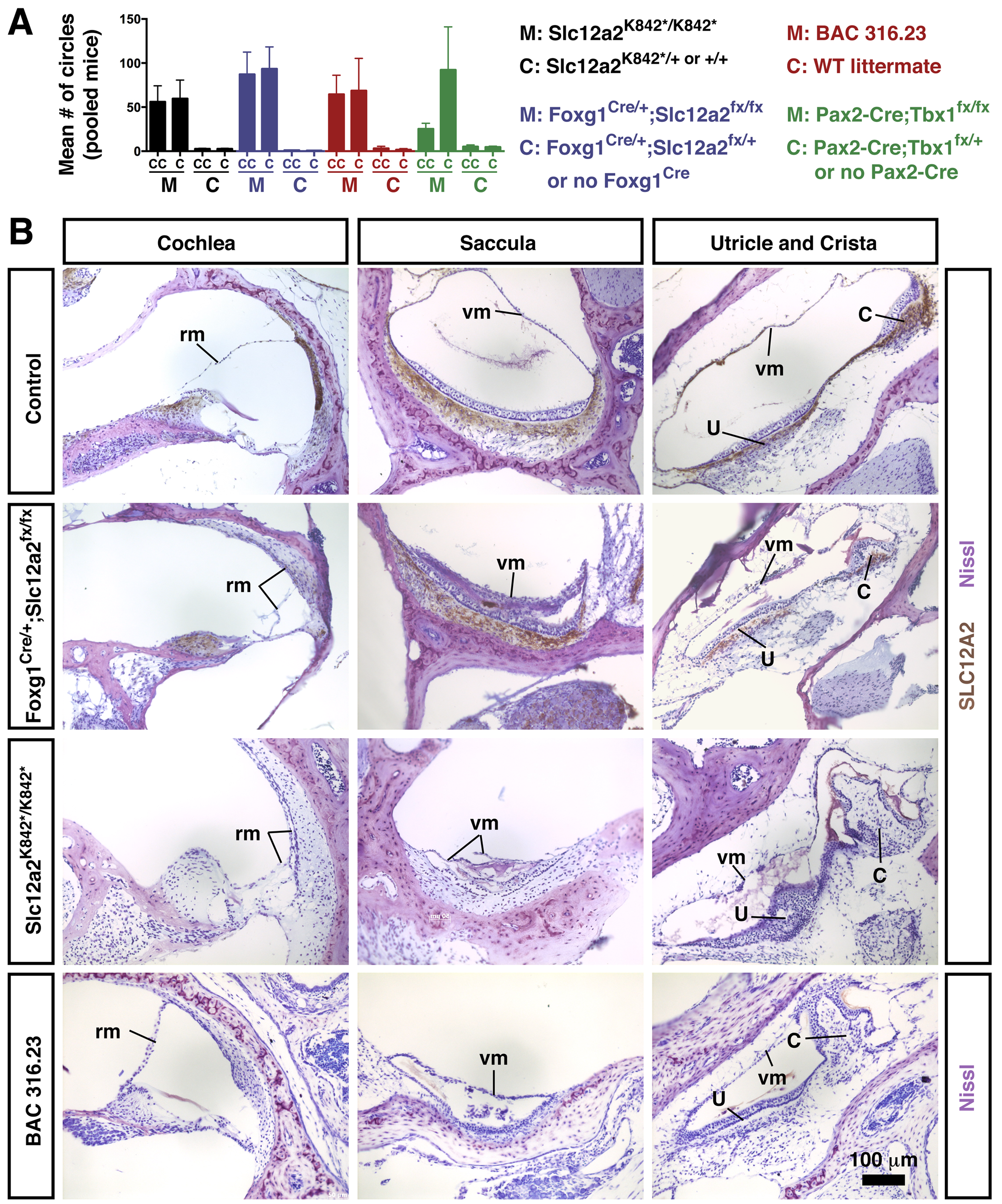

Supplement: S1 Fig — (A) Automated quantification of spontaneous circling behavior during open field locomotor activity confirms that several mutant mouse lines (M) with genetically caused inner ear defects circle, whereas littermate controls (C) do not. Slc12a2K842*/K842* and controls, n = 49, 20; Foxg1Cre/+;Slc12a2fx/fx and controls, n = 23, 15; BAC 316.23 and controls, n = 10, 6; Pax2-Cre;Tbx1fx/fx and controls, n = 4, 6. Mean ± SEM. (B) Immunohistochemical staining for SLC12A2 (Brown; except for BAC 316.23) of inner ear sections reveal morphological defects: rm collapse in the cochlea, vm collapse in the saccula, and degeneration of the utricle and cristae in Foxg1Cre/+;Slc12a2fx/fx,Slc12a2K842*/K842* and BAC 316.23 mutants. Nissl counterstain (purple). BAC, bacterial artificial chromosome; C, cristae; rm, Reissner’s membrane; SEM, standard error of the mean; U, utricle; vm, vestibular membrane. (TIF) [file pbio.2002988.s004.tif]

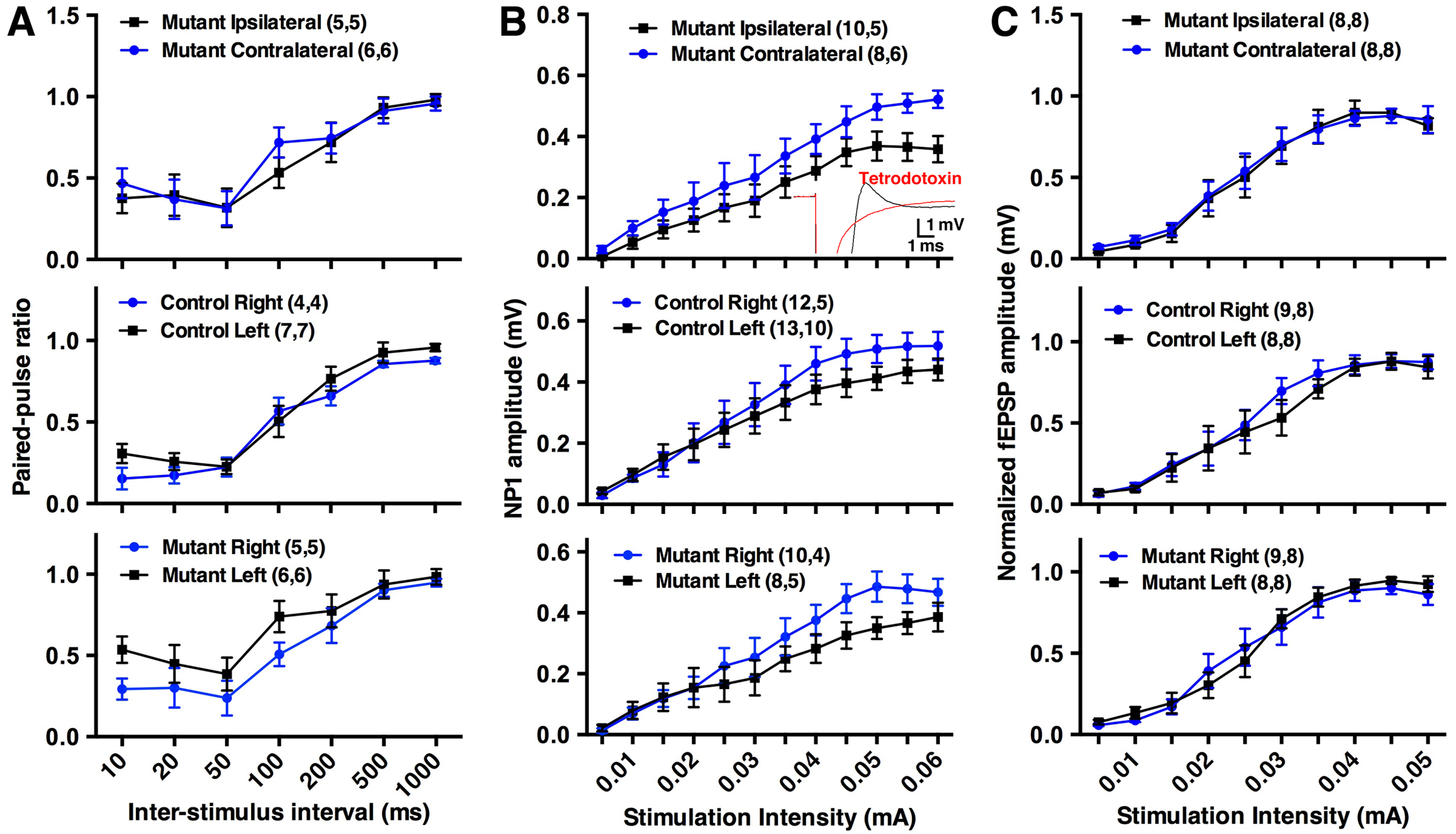

Supplement: S2 Fig — (A) No differences were detected in presynaptic function as indicated by paired-pulse ratios (slope of fEPSP no. 2/slope fEPSP no. 1) at 0.05 mA stimulation intensity when comparing the contralateral and ipsilateral hemispheres of Slc12a2K842*/K842*mutants (p = 0.67), the left or right hemispheres of littermate controls (p = 0.52), or the left or right hemispheres of Slc12a2K842*/K842* mutants (p = 0.58). (B) As a measure of neuronal excitability, the amplitude of the action potential component (NP1) of the fEPSP across the range of stimulation intensities was similar between hemispheres in all comparisons. Inset illustrates the presynaptic fiber volley (NP1; black trace) and its sensitivity to tetrodotoxin, a voltage-gated sodium channel inhibitor (red trace) with the EPSP previously blocked with NBQX and AP5. Mean ± SEM, two-way repeated measures ANOVA. (C) No significant difference in the slope of the input-output curves normalized to the peak response was detected between hemispheres in all comparisons. Numbers in parentheses indicate the total number of recordings followed by the number of mice. EPSP, excitatory postsynaptic potential; fEPSP; field excitatory postsynaptic potential; NP1, negative peak 1; SEM, standard error of the mean. (TIF) [file pbio.2002988.s005.tif]

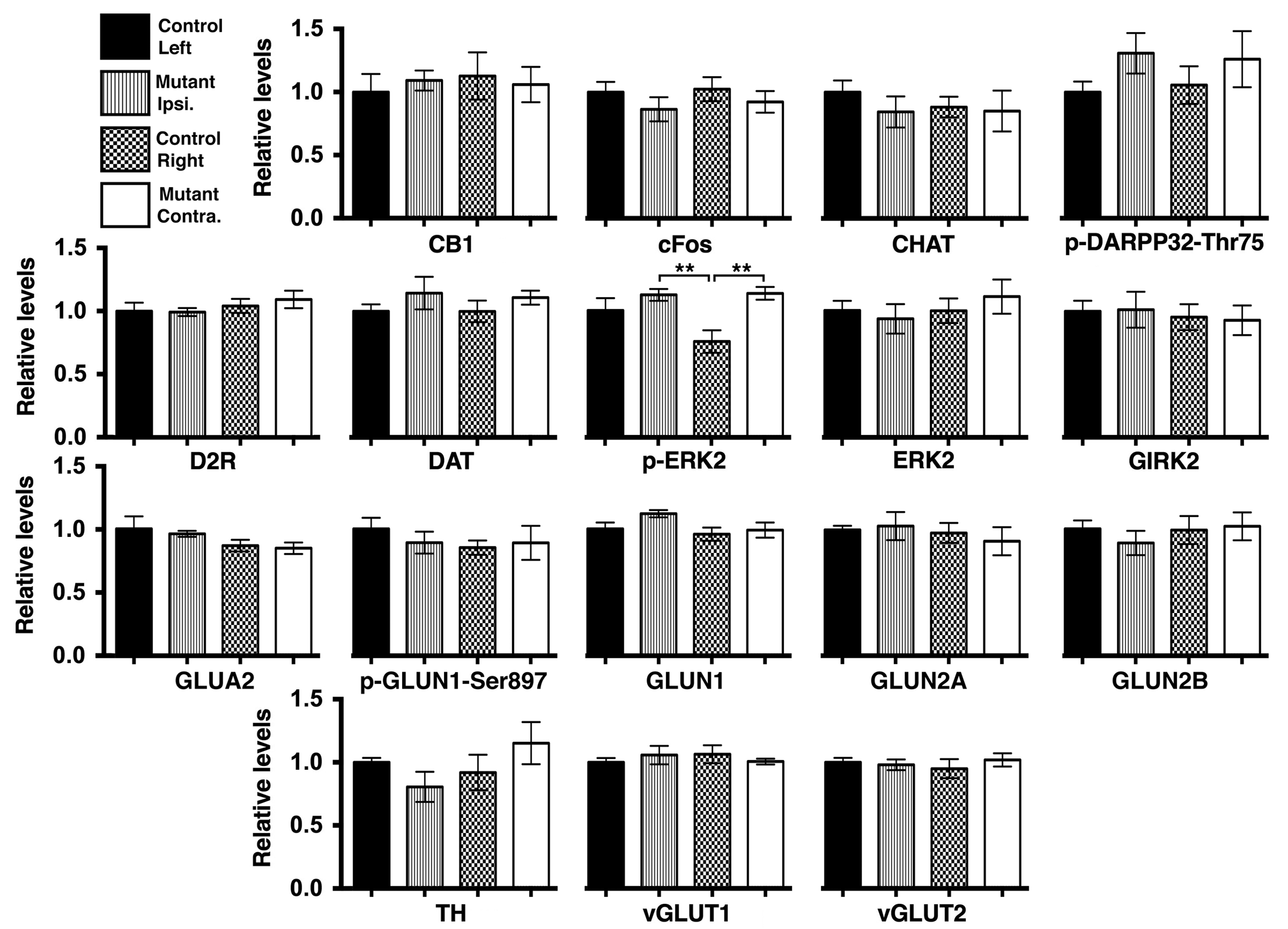

Supplement: S3 Fig — Quantification of western blots on striatal lysates from Slc12a2K842*/K842* mutants and littermate controls. Values are normalized to β-actin. n = 6 mice/genotype (except n = 3 for p-GLUN1-Ser897). Two-tailed unpaired t test. For p-ERK2 control right vs mutant contralateral, p = 0.0039, and control right versus mutant ipsilateral p = 0.0044. CB1, cannabinoid receptor type 1; CHAT, choline acetyltransferase; D2R, dopamine receptor-2; DAT, dopamine transporter; GIRK2, G protein-coupled inwardly-rectifying potassium channel-2; GLUA2, α-amino-3-hydroxy-5-methyl-4-isoxazolepropionic acid receptor (AMPAR) subunit; GLUN1,GLUN2A,GLUN2B, N-methyl-D-aspartate receptor (NMDAR) subunits; p-ERK, phosphorylated extracellular signal-regulated kinase; TH, tyrosine hydroxylase; vGLUT1,vGLUT2, vesicular glutamate transporter-1 and 2. (TIF) [file pbio.2002988.s006.tif]

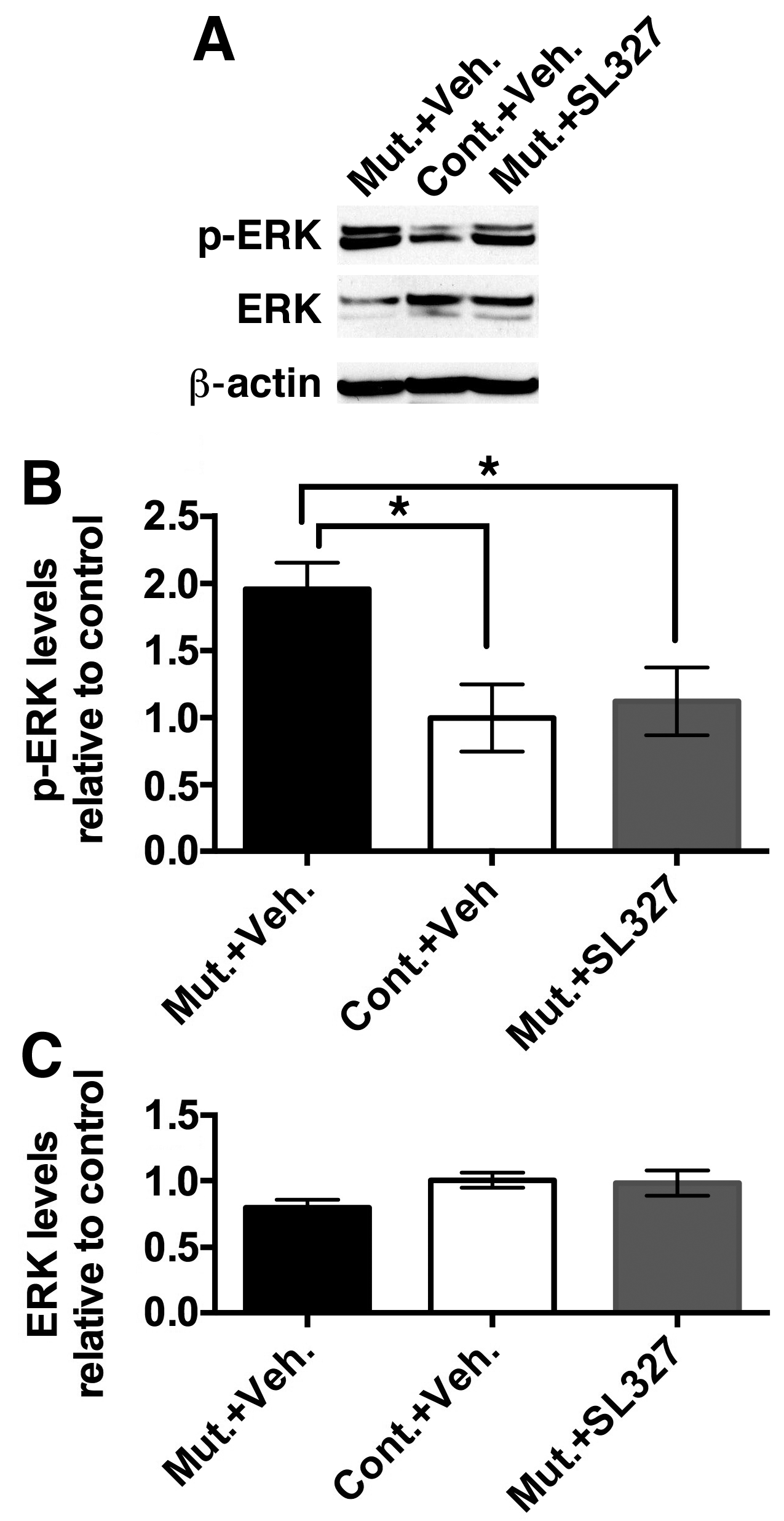

Supplement: S4 Fig — (A) Western blot analysis of striatal lysates fromSlc12a2K842*/K842* mutants receiving the MEK inhibitory SL327 or vehicle and controls receiving vehicle. (B) As expected, mutant mice treated with vehicle (n = 6) had elevated levels of p-ERK compared to controls treated with vehicle (n = 4) (p = 0.038). However, levels of p-ERK are reduced in mutants receiving SL327 (n = 10) compared with mutants receiving vehicle (p = 0.038), such that the levels in mutants receiving SL327 approach those found in control mice treated with vehicle (p = 0.95). Mean ± SEM, one-way ANOVA with Tukey’s multiple comparisons test. (C) Levels of unphosphorylated ERK were not affected by SL327 administration. ERK, extracellular signal-regulated kinase; MEK, mitogen activated protein kinase kinase; p-ERK, phosphorylated extracellular signal-regulated kinase; SEM, standard error of the mean. (TIF) [file pbio.2002988.s007.tif]

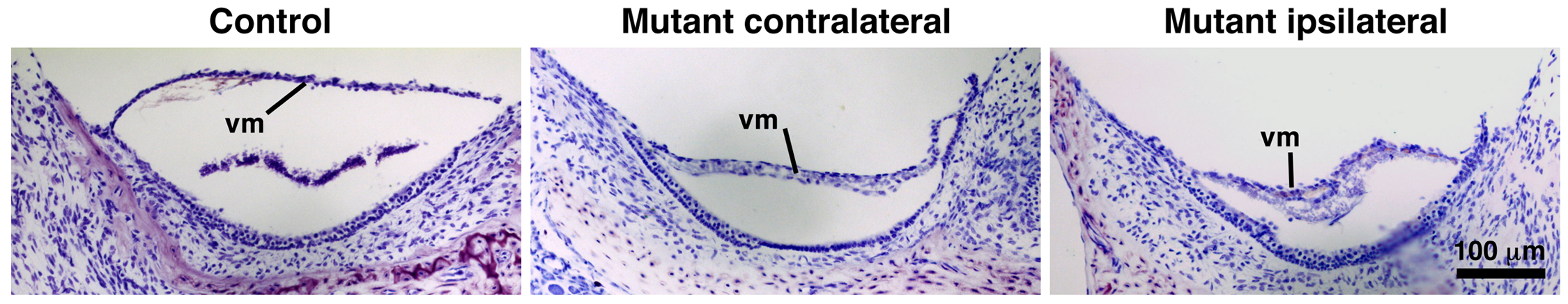

Supplement: S5 Fig — Nissl stained sections through the saccula shows uneven levels of vm collapse between the contralateral and ipsilateral ear of Tbx1Cre/+;Slc12a2fx/fx mutants (n = 5), prior to complete vestibular failure. vm, vestibular membrane. (TIF) [file pbio.2002988.s008.tif]
